# Utilization and Impact of Pharmacist-led, Urinary Culture Follow-Up After Discharge from the Emergency Department

**DOI:** 10.5811/westjem.59116

**Published:** 2023-05-03

**Authors:** Danny Pham, Stephen Lee, Sadaf Abrishami, Bharath Chakravarthy, Soheil Saadat

**Affiliations:** * University of California Irvine Medical Center, Department of Pharmacy, Orange, California; † University of California Irvine Medical Center, Department of Emergency Medicine, Orange, California

## Abstract

**Introduction:**

Urinary tract infections (UTI) are a common reason for an emergency department (ED) visit. The majority of these patients are discharged directly home without a hospital admission. After discharge, emergency physicians have traditionally managed the care of the patient if a change is warranted (as a result of urine culture results). However, in recent years clinical pharmacists in the ED have largely incorporated this task into their standard practice. In our study, we aimed to 1) describe our unique process in having a pharmacist-led, urinary culture follow-up, and 2) compare it to our previous, more traditional process.

**Methods:**

In our retrospective study, we evaluated the impact of a pharmacist-led, urinary culture follow-up program after discharge from the ED. We included patients prior to and after the implementation of our new protocol to compare the differences. The primary outcome was time to intervention after urine culture result was released. Secondary outcomes included rate of documentation of intervention, appropriate interventions made, and repeat ED visits within 30 days.

**Results:**

We included a total of 265 unique urine cultures from 264 patients in the study: 129 cultures were from the period prior to implementation of the protocol, and 136 were from the post-implementation period. There were no significant differences between pre- and post-implementation groups for the primary outcome. Appropriate therapeutic intervention based on positive urine culture results was 16.3% in the pre-implementation group vs 14.7% in the post-implementation group (P=0.72). Secondary outcomes of time to intervention, documentation rates, and readmissions were similar between both groups.

**Conclusion:**

Implementation of a pharmacist-led, urinary culture follow-up program after discharge from the ED led to similar outcomes as a physician-run program. An ED pharmacist can successfully run a urinary culture follow-up program in an ED without physician involvement.

## INTRODUCTION

Urinary tract infections (UTI) are the most common outpatient infections in the United States with over 10 million cases annually.^1^ In the ED, UTIs account for two million annual visits.^2^ Of this group, the majority of these patients are discharged directly home from the ED.

For patients with a UTI, urine cultures are obtained so that clinicians can compare antimicrobial species and antibiotic susceptibility to selected treatment. Patients are discharged from the ED with empiric antibiotics (based on institution treatment algorithms) while the results of the urine culture are processing. Once the cultures are finalized, standard practice is to follow up with the patient if medical therapy modification is required. For example, if the patient received an antibiotic that is resistant on culture susceptibilities, a phone call would have to be made to access a change in therapy. With the increasing resistance rates of antibiotics and development of multi-drug-resistant organisms, more patients have been requiring antibiotic therapy modification after culture results.^3^

Although most institutions provide discharge culture follow-up, there is not a standardized workflow for this common practice.^4^–^9^ Traditionally, the emergency physician would get notified of discharge culture results and would have to make therapy modifications. The physicians may have worked in conjunction with nurses, nurse practitioners, physician assistants, or pharmacists to triage culture results.^4^–^9^ However, our new process would allow the clinical pharmacist the independence to access and modify urine culture results under a specified collaborative protocol.

Clinical pharmacists in the ED, especially those who have done residency training, are capable of interpreting culture results and identifying the optimal antibiotics. Studies involving pharmacists in discharge culture follow-up have shown a decrease in ED revisits and hospital admissions.^10^–^11^ Although there is evidence supporting pharmacist involvement, data is specifically lacking for a pharmacist-led program without physician consultation. At our institution, we implemented a pharmacist-led, urinary culture follow-up protocol for patients discharged from the ED. Under the stipulations of the protocol, pharmacists in the ED were empowered to interpret and intervene of their own volition. In this study our aim was to assess the efficacy of this protocol in providing timely and appropriate therapeutic interventions for this patient population while describing our unique process.

## METHODS

We report a retrospective study on the impact of a pharmacist-led, urinary culture follow-up after discharge from the ED. This study was conducted at the University of California, Irvine Medical Center. The ED has over 50,000 patient visits annually with ≈50 patients discharged per week with a diagnosis of a UTI. Three pharmacists provided decentralized services in the department for 16 hours on weekdays and eight hours on the weekends.

In March 2020, a pharmacist-led, urine culture follow-up protocol was implemented. Prior to implementation of the protocol, ED pharmacists assisted emergency physicians in reviewing cultures and could provide recommendations regarding treatment but required physician authorization before making changes. The pharmacist would have to approach an attending physician who was on shift to discuss the culture results. With the implementation of the new protocol, pharmacists were privileged to independently adjust antibiotic regimens based on urine culture results. The ED pharmacists were able to add, adjust, and discontinue antibiotics within the specifications of the protocol.

Based on our protocol, if an intervention was required, the pharmacist would contact the patient to conduct an interview. In doing so, the pharmacist would assess the patient’s condition, medication compliance, and treatment efficacy to decide whether any interventions would be required. If the patient required a medication change, the pharmacist would notify the patient of the new treatment plan and provide counseling/education. The pharmacist would send a new prescription and document the intervention made on the patient’s electronic health record. Lastly, the pharmacist would notify the original prescriber of the updated treatment plan. Pharmacists in the ED would take about 20 minutes a day to review urine culture results. On average, there were about 10 cultures a day to review, with most of them not needing an intervention. There were no direct costs associated with implementation of this program.

We included patient data from two months before and after implementation. Patients were included in the study if they were ≥18 years and seen in the ED with a urine culture collected. Patients were excluded from the study if they were admitted to the hospital. We included patients treated after the new protocol was implemented. The control group was composed of patients prior to the protocol implementation.

The primary outcome was time to intervention after culture results were released. Time to intervention was measured from time of culture result to when a progress note was charted regarding the result. Secondary outcomes included rate of documentation of intervention, rate of appropriate interventions made, and repeat ED visits within 30 days. We defined an appropriate intervention as a correct treatment plan dependent on the patient’s urine culture, which included antibiotic choice, dosing, and duration. For our statistical analysis, we used chi-squared tests for nominal data and Student *t*-tests for continuous variables. A *P*-value of less than 0.05 was considered to indicate statistical significance.

## RESULTS

A total of 265 positive urine culture results from 264 unique patients were included in the final analysis from February–May 2020: 129 culture results were from the pre-implementation period, and 136 were from the post-implementation period. Baseline characteristics were similar between both groups (Table 1). The most frequent comorbidities were immunocompromised state (8.7%), pregnancy (7.9%), and recent UTI (6.8%). Of the patients with a positive urine culture result, only 106 (40.2%) had a presentation consistent with a UTI. Of these patients, there was not a significant difference in rate of treatment-organism discordance, defined as inappropriate treatment based on the organism(s) that grew out (*P*=0.66).

The primary outcome of time to intervention was 14.5 hours in the pre-group vs 7.0 hours in the post-group (*P*=0.54). For the secondary outcomes, we found no significant differences between the pre- and post-implementation groups. Of the interventions, 8 (38.1%) vs 12 (60%) of them were documented for the pre-implementation and post-implementation groups, respectively (*P*=0.16). The rate of appropriate therapeutic interventions based on positive urine culture results was 16.3% in the pre-implementation group vs 14.7% in the post-implementation group (*P*=0.72). There was also no significant difference in repeat ED visits within 30 days (Table 2). The initial prescribing physicians were notified of any interventions made by pharmacists, and the interventions were deemed appropriate after being reviewed by the physicians. Appropriate interventions were defined as antibiotics at discharge being susceptible based on urine culture results.

## DISCUSSION

In this retrospective study assessing the efficacy of a pharmacist-led, urine culture follow-up protocol, we found no significant difference in the time to intervention of urine culture results of patients with UTI discharged from the ED. None of the secondary outcomes showed a statistically significant difference pre- and post-implementation of this protocol. Despite not requiring direct physician oversight, intervention rates and repeat ED visits were similar after protocol implementation. This study provides evidence that pharmacists working independently are capable of appropriately managing urine cultures. Although our study did not show these results, having a pharmacist manage cultures could potentially increase documentation rates, decrease time to intervention, and decrease readmissions.

Like previous studies, our study described implementation of a new process for managing ED discharge cultures and compared post-implementation data with pre-implementation data.^12^–^13^ Having pharmacists work on discharge cultures is not unique to the ED setting. However, our protocol privileged ED pharmacists to work independently to review and manage discharge urine cultures. Prior to our protocol implementation, ED pharmacists were already involved in reviewing discharge culture results. Pharmacists were able to identify when interventions were required and would advise an attending physician on call to make an intervention. The difference in protocol implementation is that now pharmacists conduct interventions independently, which may allocate more time for emergency physicians to manage more acute patients. Despite a pharmacist solely managing these interventions, there was not a drop-off in appropriate interventions.

A potential benefit of an ED pharmacist-led protocol is the capability to reduce time to intervention. Because the initial prescribing physician was not involved in the management of culture callbacks, there were no delays due to physician staffing schedules. Furthermore, current physicians who were staffing in the department did not need to be notified of past culture results and then address them. This in turn would free up more time for direct patient care. Additionally, the pharmacist did not have to wait on an ED clinician to discuss the culture result, as required by many pharmacy-led protocols, and could intervene more quickly of their own volition under the collaborative practice. Although our study did not show it, our protocol could potentially lead to faster time to intervention and could identify treatment discordances and inappropriate treatment of UTIs, which would in turn reduce treatment failures, antimicrobial resistance, and readmissions.

## LIMITATIONS

A limitation in our study included the short time frame of data collection. The study data was only collected for four months, and the results could have been more robust with a longer collection period and greater sample size. A power analysis was not done; so it is unknown whether the study was adequately powered to detect a difference. Another limitation is the retrospective study design, and so we could not control for other confounding variables. A delay or lack of documentation could affect the time-to-intervention results.

## CONCLUSION

This study describes the implementation of a pharmacist-led, urinary culture follow-up protocol in the ED and demonstrates that ED pharmacists can successfully lead urine culture follow-ups without physician consultation under a collaborative practice. We found no significant differences in time to intervention after urine culture result was released, nor in appropriate interventions made or repeat ED visits within 30 days. The protocol described here could be implemented in other institutions and expanded upon to provide more opportunities for pharmacist clinical services.

## Figures and Tables

**Table 1:** Baseline demographics and clinical characteristics according to cohort.

Characteristics	Pre-group, n = 129	Post-group, n = 136	P-value
Female, n (%)	111 (86.1)	118 (86.8)	0.87
Age, mean ± SD	48.5 ± 20.8	47.4 ± 19.9	0.68
Clinical comorbidities,a (%)	38 (29.5)	51 (37.5)	0.17
Pregnancy, n (%)	11 (8.5)	10 (7.4)	0.72
Recent UTI, n (%)	6 (4.7)	12 (8.8)	0.18
Nephrostomy tube, n (%)	1 (0.8)	4 (2.9)	0.37
Immunocompromised, n (%)	7 (5.4)	16 (11.8)	0.07
History of MDR organisms, n (%)	1 (0.8)	1 (0.7)	>0.99
Recent urological procedure, n (%)	3 (2.3)	1 (0.7)	0.36
Catheterized, n (%)	6 (4.7)	11 (8.1)	0.25
Neurological handicaps, n (%)	5 (3.9)	8 (5.9)	0.45
Positive urine analysis, n (%)	63 (48.8)	78 (57.4)	0.17
Received antibiotics in ED, n (%)	39 (30.2)	55 (40.4)	0.08
Positive urine culture growth, n (%)	-	-	0.20
Single pathogen, n (%)	93 (72.1)	88 (64.7)	-
Multiple pathogens, n (%)	36 (27.9)	48 (35.3)	
MDR pathogens, n (%)	7 (5.4)	10 (7.4)	>0.99
ESBL, n (%)	6 (85.7)	9 (90)	-
MRSA, n (%)	1 (14.3)	1 (10)	
Rate of treatment-organism discordance a	-	-	0.66
Yes, n (%)	15 (25.9)	16 (22.5)	-
No, n (%)	43 (74.1)	55 (77.5)	

a After removal of colonization and asymptomatic patients.

*UTI*, urinary tract infection *ED*, emergency department; *MDR*, multidrug resistant; *ESBL*, extended-spectrum beta-lactamases; *MRSA*, methicillin-resistant *Staphylococcus aureus*.

**Table 2:** Discharge outcomes and associated interventions according to cohort.

Characteristics	Pre-group, n = 129	Post-group, n = 136	P-value
Time to intervention, median [IQR]	14.5 [2.7–25.7]	7.0 [2.3–15.7]	0.54
Discharged with antimicrobials, n (%)	60 (46.5)	70 (51.5)	0.42
Interventions required, n (%)	21 (16.3)	20 (14.7)	0.72
Interventions documented, n (%)	8/21 (38.1)	12/20 (60)	0.16
Start new antibiotics, n (%)	1 (12.5)	2 (16.7)	
Change in antibiotics, n (%)	6 (75)	6 (50)	-
Discontinue antibiotics, n (%)	1 (12.5)	4 (33.3)	
Re-admitted within 30 days, n (%)	11 (8.5)	2 (2.3)	0.08

*IQR*, interquartile range.
